# Elevated β-catenin pathway as a novel target for patients with resistance to EGF receptor targeting drugs

**DOI:** 10.1038/srep13076

**Published:** 2015-08-13

**Authors:** Asuka Nakata, Ryo Yoshida, Rui Yamaguchi, Mai Yamauchi, Yoshinori Tamada, Andre Fujita, Teppei Shimamura, Seiya Imoto, Tomoyuki Higuchi, Masaharu Nomura, Tatsuo Kimura, Hiroshi Nokihara, Masahiko Higashiyama, Kazuya Kondoh, Hiroshi Nishihara, Arinobu Tojo, Seiji Yano, Satoru Miyano, Noriko Gotoh

**Affiliations:** 1 Division of Cancer Cell Biology, Cancer Research Institute, Kanazawa University; 2 Division of Molecular Therapy, Institute of Medical Science, University of Tokyo; 3 The Institute of Statistical Mathematics, 10-3 Midori-cho, Tachikawa Tokyo, 190-8562, Japan; 4 Laboratory of Sequence Analysis, Institute of Medical Science, University of Tokyo; 5 Laboratory of DNA information Analysis, Institute of Medical Science, University of Tokyo; 6 Department of Surgery, Tokyo Medical University; 7 Department of Respiratory Medicine, Graduate School of Medicine, Osaka City University; 8 Division of Internal Medicine and Thoracic Oncology, National Cancer Center Hospital; 9 Department of Thoracic Surgery, Osaka Medical Center for Cancer and Cardiovascular Diseases; 10 Department of Thoracic, Endocrine Surgery and Oncology, Institute of Health Bioscience, The University of Tokushima Graduate School; 11 Laboratory of Translational Pathology, Hokkaido University Graduate School of Medicine; 12 Division of Medical Oncology, Cancer Research Institute, Kanazawa University.

## Abstract

There is a high death rate of lung cancer patients. Epidermal growth factor receptor tyrosine kinase inhibitors (EGFR-TKIs) are effective in some lung adenocarcinoma patients with EGFR mutations. However, a significant number of patients show primary and acquire resistance to EGFR-TKIs. Although the Akt kinase is commonly activated due to various resistance mechanisms, the key targets of Akt remain unclear. Here, we show that the Akt-β-catenin pathway may be a common resistance mechanism. We analyzed gene expression profiles of gefitinib-resistant PC9M2 cells that were derived from gefitinib-sensitive lung cancer PC9 cells and do not have known resistance mechanisms including EGFR mutation T790M. We found increased expression of *Axin*, a β-catenin target gene, increased phosphorylation of Akt and GSK3, accumulation of β-catenin in the cytoplasm/nucleus in PC9M2 cells. Both knockdown of β-catenin and treatment with a β-catenin inhibitor at least partially restored gefitinib sensitivity to PC9M2 cells. Lung adenocarcinoma tissues derived from gefitinib-resistant patients displayed a tendency to accumulate β-catenin in the cytoplasm. We provide a rationale for combination therapy that includes targeting of the Akt-β-catenin pathway to improve the efficacy of EGFR-TKIs.

Lung cancer is the leading cause of cancer death worldwide^1^. Lung cancer is divided into two histological subtypes, small cell lung cancer (SCLC) and non-small cell lung cancer (NSCLC). NSCLC is further subdivided into three groups, adenocarcinoma, squamous carcinoma and large cell carcinoma. The traditional chemotherapies for NSCLC patients have limited efficacy^2^,^3^,^4^ as well as treatment-related toxicity^5^,^6^. Therefore, more effective and more specific therapies for each NSCLC patients are greatly needed. Adenocarcinoma is the most common subtype of NSCLC and is frequently associated with the genetic alteration of epidermal growth factor receptor (EGFR), such as deletion in exon 19, L858R mutation or wild-type EGFR amplification^7^. Many NSCLC patients who harbor mutations in the tyrosine kinase domain of the EGFR have been reported to show good response to EGFR tyrosine kinase inhibitors (EGFR-TKIs)^8^,^9^,^10^. The EGFR-TKIs such as gefitinib or elrotinib are pioneer molecular targeted drugs that have been used as the first-line treatment for patients with EGFR mutations^11^,^12^.

However, one serious problem that has arisen is that patients who initially had a good response to EGFR-TKIs ultimately experience a recurrence within only a few years. Several mechanisms of such acquired resistance to EGFR-TKIs have been reported. A secondary mutation of the EGFR, T790M, which impairs the binding between EGFR and gefitinib, was observed in many patients with acquired resistance^13^,^14^,^15^. Amplification of the MET tyrosine kinase^16^,^17^ and overexpression of HGF^18^,^19^, a ligand for MET, are also associated with acquired resistance to EGFR-TKIs. Increased expression of N-cadherin is a further potential mechanism of resistance to EGFR-TKIs^20^. There are additionally a significant number of patients who harbor activating mutations in the EGFR but who are primarily resistant to EGFR-TKIs. Similar to the situation in acquired resistance, elevation of the HGF-MET pathway can occur in primarily resistant lung adenocarcinoma tissues^21^. It is known that activity of the Akt kinase is commonly upregulated in cancer cells that are resistant to EGFR-TKIs^7^. However, the mechanisms that underlie resistance to EGFR-TKIs are still unclear and there are many patients for whom the appropriate molecular targets for therapy are unknown.

Activation of the Wnt/β-catenin pathway has been observed in a variety of cancers^22^. Wnt binds to a cell surface receptor complex that consists of the Frizzled (FZD) protein and the low density lipoprotein receptor-related protein (LRP)^23^. These receptors transduce a signal from Wnt to the intracellular proteins Disheveled (Dsh), glycogen synthetase kinase 3 (GSK3), Axin, Adenomatous Polyposis Coli (APC) and β-catenin. In the steady state, membrane β-catenin is bound to α−catenin and E-cadherin. On the other hand, cytoplasmic β-catenin is phosphorylated by GSK3 and phosphorylated β-catenin is degraded by the ubiquitin-protease system. GSK3 activity thereby maintains cytoplasmic β-catenin expression at a low level. Thus, phosphorylation of β-catenin by GSK3 tightly regulates the cytoplasmic pool of β-catenin^22^. Wnt stimulation promotes the phosphorylation of GSK3, which suppresses the catalytic activity of GSK3 and leads to the stabilization of β-catenin. β-catenin in the cytoplasm shuttles into the nucleus where it forms a complex with the TCF/LEF family of transcription factors, leading to induction of β-catenin/TCF-dependent transcription. Mutations and amplification of Wnt signaling molecules have been observed in many types of cancer. In NSCLC, although APC mutation has rarely been observed^24^, it has been reported that Wnt ligand and Fzd are overexpressed^25^,^26^,^27^ and that Wnt antagonists are downregulated^28^,^29^,^30^.

In this study, we established gefitinib-resistant cell lines by long-term exposure of a gefitinib-sensitive cell line to gefitinib to explore novel mechanisms of resistance to EGFR-TKIs. Comparison of the gene expression pattern of gefitinib-resistant cell lines with that of the parental cell lines showed that Wnt/β-catenin signaling molecules were upregulated in the gefitinib-resistant cell lines. Furthermore, we demonstrated that sensitivity to gefitinib was associated with β-catenin activation. Our data suggest that combination therapy of β-catenin pathway-targeting drugs and EGFR-TKIs will be effective for adenocarcinoma patients with primary or acquired resistant to EGFR-TKIs. Our finding raises the intriguing possibility that activation of the Akt-β-catenin pathway might be a common mechanism for conferring resistance to cancer treatment, not only to EGF-TKIs, but also to other types of treatment, including chemotherapy and radiotherapy.

## Results

### Establishment of gefitinib-resistant PC9M2 cells from PC9 cells harboring gefitinib-sensitive mutations in the EGFR

**Figure 1:**
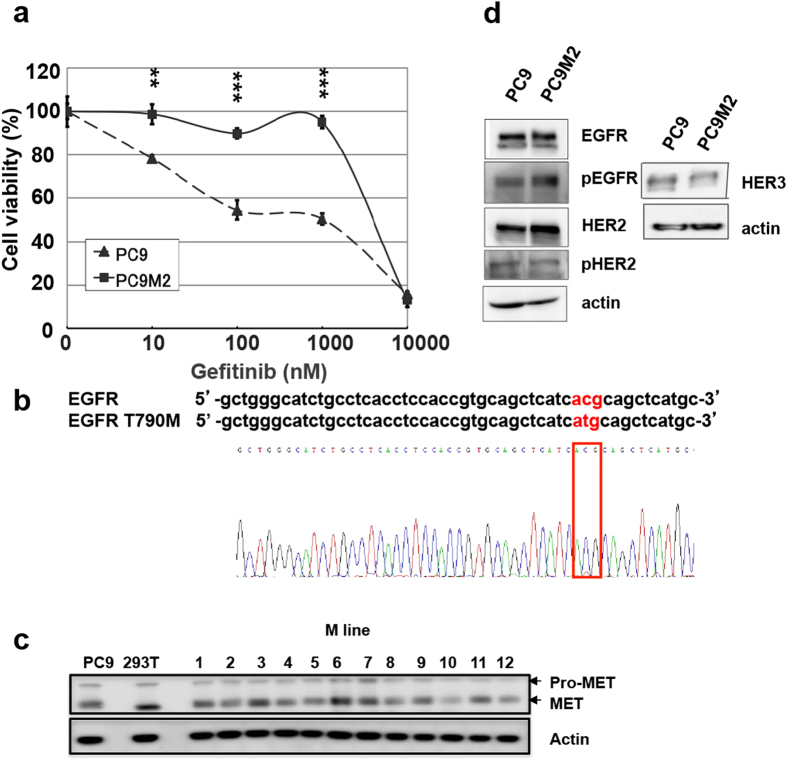
Establishment of gefitinib-resistant PC9M2 cells from PC9 cells. (**a**) Parental PC9 cells and PC9M2 cells were grown for 72 h in RPMI1640 with or without gefitinib at the indicated concentrations. Cell viability was measured using the MTT assay. The data are represented as mean ± SD, N = 4. ^**^P < 0.01 and ^***^P < 0.001. (**b**) DNA sequencing analysis to check for the presence of a secondary mutation in the EGFR in PC9M2 cells. (**c**) Western blotting of **c**ell lysates from parental PC9, from the M line of PC9 gefitinib-resistant clones including PC9M2 and from HEK293T cells, probed with anti-MET and anti-actin (loading control) antibodies. Pro-MET, precursor of MET. These cropped blots used in this figure were run under the same experimental conditions. (**d**) Western blotting of lysates of PC9 and PC9M2 cells, which were prepared from cells in the steady state, using anti-EGFR, anti-HER2, anti-HER3 and actin (loading control) antibodies. Experiments were performed at least three times and representative results were presented.

Gefitinib-resistant PC9M2 cells were established by culturing gefitinib-sensitive PC9 cells, which harbor a 5 amino acid deletion in the EGFR tyrosine kinase domain^31^, in the presence of a low amount of gefitinib for several months. Parental PC9 cells or gefitinib-resistant PC9M2 cells were then treated with various concentrations of gefitinib for 72 hours and cell viability was measured using MTT assays (Fig. 1a). Following treatment with 1,000 nM (1 μM) gefitinib, almost 100% of the PC9M2 cells survived, whereas only ~50% of the parental PC9 cells survived. A secondary EGFR T790M mutation, MET amplification and overexpression of HER family members are known mechanisms of acquired or primary resistance to EGFR-TKIs^7^,^31^. We therefore determined if any of these mechanisms underlay the resistance of the PC9M2 cells to gefitinib. We performed reverse transcription (RT)-PCR and Sanger DNA sequencing of the PCR product to determine if the EGFR T790M mutation was present (Fig. 1b). PC9M2 cells, like the parental PC9 cells, did not harbor this mutation. Western blotting of the expression level of the MET protein indicated that MET protein levels were comparable in gefitinib-resistant PC9M lines including PC9M2, which means No. 2 of PC9M lines and PC9 cells (Fig. 1c). Western blotting showed a modest increase in the expression levels of EGFR, HER2 and phosphorylated EGFR in PC9M2 compared to PC9 cells, while the expression levels of HER3 and phosphorylated HER2 were comparable (Fig. 1d).

### β-catenin-induced transcription is upregulated in PC9M2 cells

**Figure 2:**
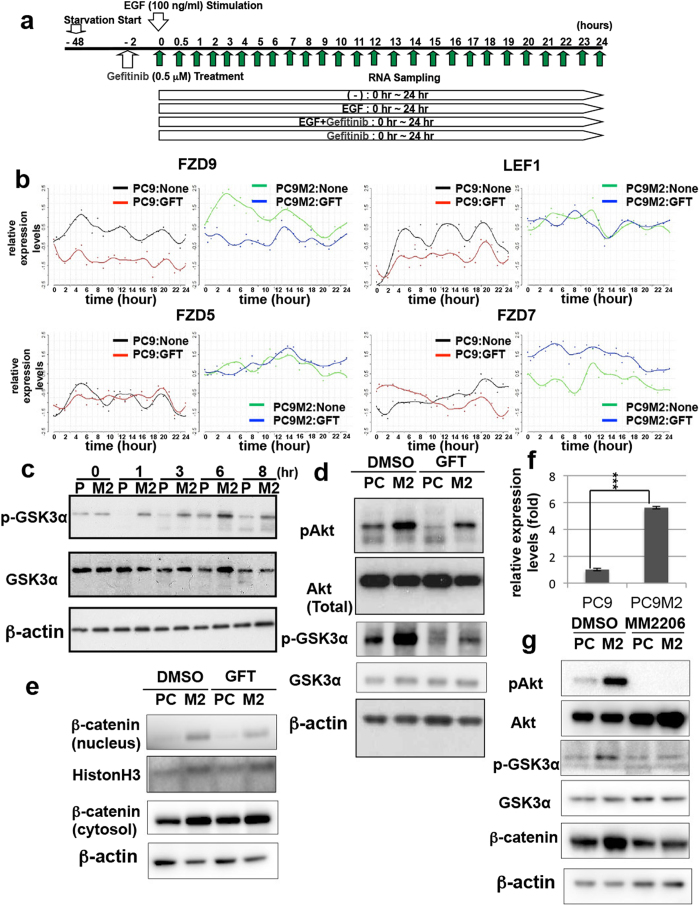
Wnt/β-catenin pathway-related genes are upregulated in PC9M2 cells. (**a**) Time course of mRNA sampling for DNA microarray analysis. PC9 and PC9M2 cells were starved for 48 h, then treated with or without gefitinib and cultured in the presence or absence of EGF. mRNA was extracted at the indicated times. (**b**) The mRNA expressions levels of the Wnt/β-catenin pathway related genes *FZD5*, *7* and *9* and *LEF* of the cells in (**a**) were plotted using DNA microarray data. None/GFT; −/+ gefitinib respectively. (**c**,**d**) Western blotting of lysates of PC9 (P) and PC9M2 (M2) cells that were treated with gefitinib (GFT) at 1 μM for the indicated times (**c**) and at 0.5 μM for 1 hr (**d**). Treatment with DMSO is a control. Blots were probed with phospho-GSK3α, GSKα, β-actin (loading control), phospho-Akt and Akt antibodies. (**e**) Western blotting of β-catenin in the nucleus and cytosol/membrane fraction of PC9 (PC) or PC9M2 (M2) cells treated with gefitinib (GFT) at 1 μM or control DMSO for 1 hr. (**f**) The expression levels of *Axin2* mRNA in PC9 and PC9M2 cells at the steady state, as measured using quantitative real-time (qRT)-PCR. The data are represented as mean ± SD, N = 4. ^***^P < 0.001 (*P* = 4.89^e–6^). (**g**) Western blotting of lysates of PC9 and PC9M2 cells that were treated with MM2206 at 10 μM for 5 hr. Blots were probed with phospho-Akt, Akt, phosphor-GSK3α, GSK3α, β-catenin and actin (loading control). (**c**–**e** and **g**) Experiments were perform**e**d at least three times and representative results were presented. These blots used in this figure were run under the same experimental conditions. Different pieces of the same protein blots or same samples with same conditions of electrophoresis and electrotransfer were used and presented.

To explore the novel mechanism of acquired resistance to EGFR-TKIs in PC9M2 cells, we comprehensively analyzed gene expression profiles of PC9 and PC9M2 cells using DNA microarray analysis. Since we assumed that gefitinib or EGF-treatment may affect the gene expression levels during the time course, we analyzed the gene expression profiles over 24 h, one cell cycle of PC9 cells. Cells were treated with gefitinib and EGF for various periods of time, prior to mRNA extraction (Fig. 2a). The mRNA was then analyzed using DNA microarrays to determine the time-course of gene expression of PC9 and PC9M2 cells. The presence of EGF did not significantly affect the overall gene expression profiles of PC9 or PC9M2 cells. This result is consistent with the fact that deletion mutations of the EGFR in PC9 and PC9M2 cells result in constitutive activation of the EGFR tyrosine kinase and that EGF-treatment does not further activate the EGFR tyrosine kinase^31^. Gefitinib treatment affected the overall gene expression profiles in both PC9 cells and PC9M2 cells (Fig. 2b). We noticed that gene expression levels were dynamically changed during the time course without any treatment, gefitinib, EGF or serum (labeled as “None” in Fig. 2b). We compared the gene expression profiles of PC9M2 cells with those of PC9 cells. We basically focused on the genes whose expression levels were elevated in the gefitinib-resistant PC9M2 cells than in parental PC9 cells, in spite of the presence or absence of gefitinib or EGF-treatment. We obtained 1,696 genes whose expression levels were substantially elevated in PC9M2 cells than in PC9 cells throughout the time course. By using Gene Ontology (GO) analysis, we found that Wnt/β-catenin pathway-related genes were enriched among the 1,696 genes. Some examples of the time-course expression profiles of the Wnt/β-catenin pathway related genes were shown in Fig. 2b. This result led us to hypothesize that the Wnt/β-catenin pathway is activated in PC9M2 cells.

To determine whether Wnt/β-catenin signaling is activated in PC9M2 cells, we first examined the phosphorylation status of GSK3α under gefitinib treatment, by Western blotting. As shown in Fig. 2c, the phosphorylation levels of GSK3α were higher in PC9M2 cells than those of PC9 cells, under gefitinib treatment. Akt is known to be one of the serine/threonine kinases that phosphorylate GSK3α^32^. Using Western blotting, we found that the phosphorylation levels of Akt were higher in PC9M2 cells than in PC9 cells (Fig. 2d). Treatment with gefitinib greatly reduced the phosphorylation of Akt in PC9 cells, whereas the phosphorylation of Akt was still maintained in PC9M2 cells even in the presence of gefitinib. These results suggest that activated Akt phosphorylates and inactivates GSK3α in PC9M2 cells even in the presence of gefitinib. Unphosphorylated β-catenin in the cytoplasm shuttles into the nucleus and forms a complex with the TCF family of transcription factors, leading to induction of β-catenin/TCF-dependent transcription. The amount of β-catenin localized in the nucleus or the cytoplasm may reflect β-catenin activity. We therefore next compared the cellular localization of β-catenin in PC9 and PC9M2 cells using cell fractionation. Cells were treated with or without gefitinib and cell lysates were fractionated by centrifugation. The cytoplasmic/membrane and nuclear fractions of the cells were then subjected to immunoblotting using anti-β-catenin antibodies. As shown in Fig. 2e, more β-catenin was accumulated in both the cytoplasmic/membrane and nuclear fractions of PC9M2 cells than of PC9 cells. These results support the possibility that β-catenin-induced transcription is more highly activated in PC9M2 cells than in PC9 cells regardless of gefitinib treatment. To further analyze this possibility, we measured the mRNA expression of *Axin2*, which is a typical target gene of β-catenin-induced transcription^33^. qRT-PCR analysis indicated that the mRNA expression levels of *Axin2* were higher in PC9M2 cells than in PC9 cells (Fig. 2f).

Next, we treated the cells with the Akt inhibitor MM2206 to block the Akt-GSK pathway. Treatment with MM2206 reduced phosphorylation of Akt in both PC9 and PC9M2 cells. Further, MM2206 treatment reduced phosphorylated GSK3α and expression of β-catenin in PC9M2, but not in PC9 cells. These results suggest that inhibition of the Akt-GSK pathway rescues an increase in β-catenin expression in PC9M2 cells (Fig. 2g).

### Down-regulation of β-catenin activity restores gefitinib sensitivity to PC9M2 cells

**Figure 3:**
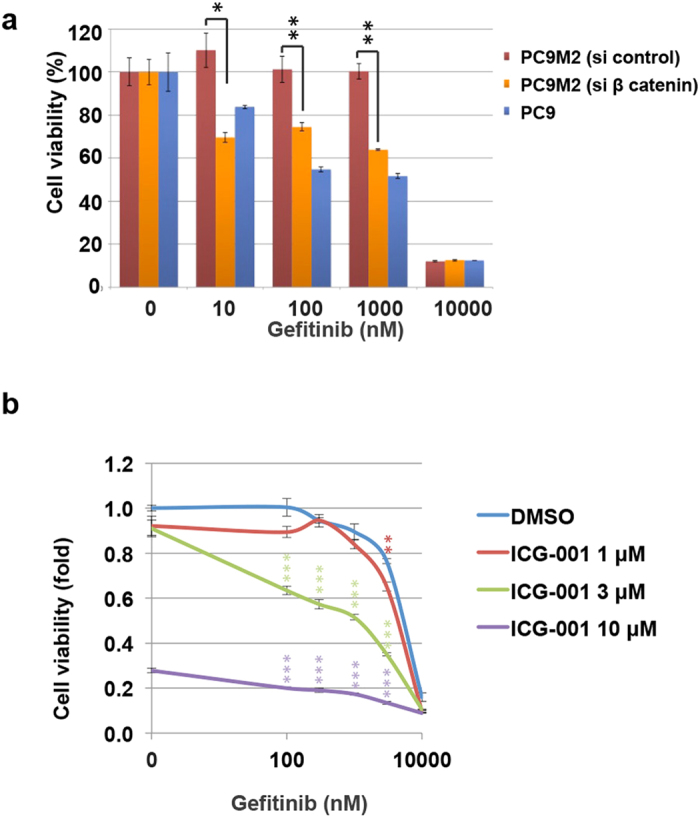
Down-regulation of β-catenin restores gefitinib sensitivity to gefitinib-resistant PC9M2 cells. (**a**) PC9M2 cells were transfected with β-catenin siRNA or control siRNA and these cells, or control PC9 cells, were treated with the indicated concentration of gefitinib for 72 h. Cell viability was determined using the MTT assay (N = 3). (**b**) PC9M2 cells were treated with the indicated concentration of ICG-001, or with control DMSO, in the presence or absence of gefitinib for 72 h. Cell viability was determined using the MTT assay (N = 3). The experiments were performed three times and the representative results were presented. The data are represented as mean ± SD. ^*^P < 0.05, ^**^P < 0.01 and ^***^P <^ ^0.01.

We next evaluated the impact of enhancement of β-catenin activity on cellular resistance to EGFR-TKIs. Gefitinib sensitivity of PC9M2 cells that were transfected with siRNAs against β-catenin or control siRNA was compared by assay of cell viability (Fig. 3a). Gefitinib sensitivity of β-catenin knockdown PC9M2 cells was increased compared to the control siRNA-transfected cells and was as high as that of the parental PC9 cells. We next assayed the effect of ICG-001, a specific inhibitor of β-catenin-TCF transcriptional activity^34^, on the gefitinib sensitivity of PC9M2 cells. ICG-001 inhibition of β-catenin activity in PC9M2 cells induced sensitivity to gefitinib in a dose-dependent manner (Fig. 3b). These data suggest that activation of β-catenin in PC9M2 cells conferred cellular resistance to gefitinib.

### Cytoplasmic localization of β-catenin is observed in tumors derived from PC9M2 cells *in vivo*

**Figure 4:**
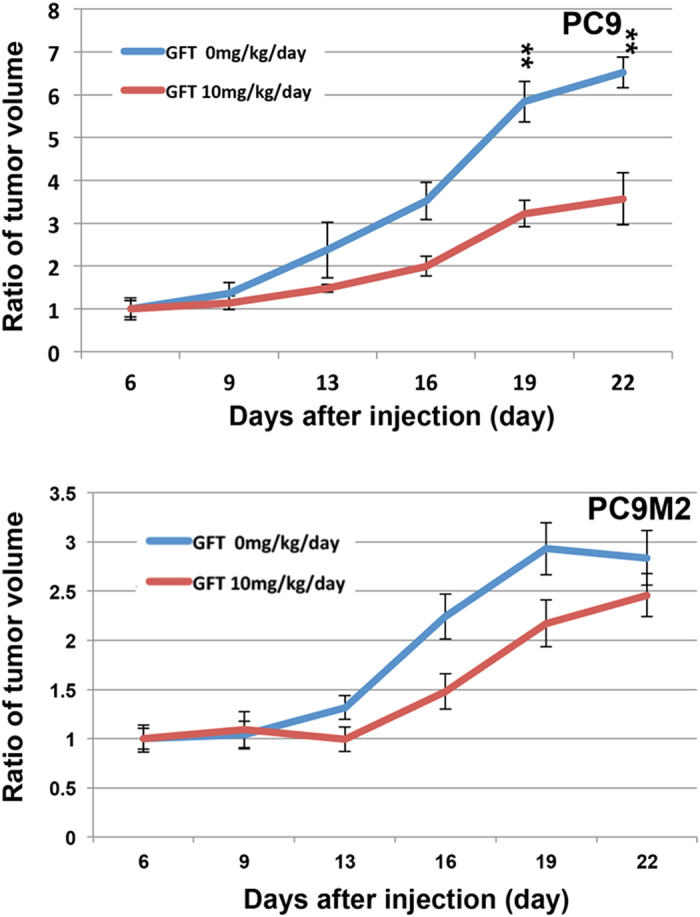
PC9M2 cells are resistant to gefitinib *in vivo*. PC9 cells (upper graph) or PC9M2 cells (lower graph) were injected into the flanks of SCID mice. Oral administration of gefitinib (0 or 10 mg/kg/day) was started at 6 days after injection. Data were represented as mean ± SD (N = 6). ^**^P < 0.01.

**Figure 5:**
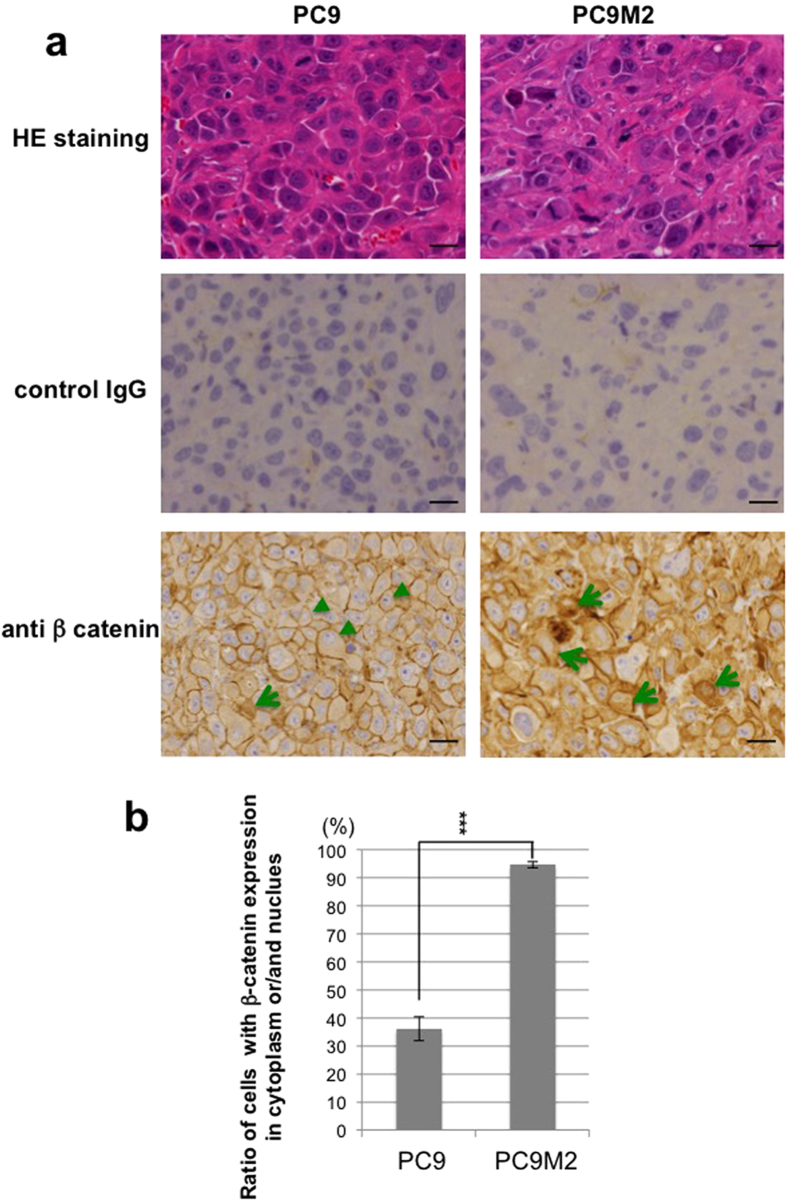
β-catenin activity may be associated with tumor sensitivity to gefitinib *in vivo*. (**a**) Parental PC9 or gefitinib resistant PC9M2 cells were injected into the flanks of SCID mice. The tumors derived from each cell type were harvested 21 days after cell injection. HE staining and immunohistochemical analysis of β-catenin expression and control IgG in representative tumors are shown. Scale bar = 20 μm. Cells with cytoplasm/nucleus staining (arrows) and cells with only plasma membrane staining (arrowheads) are indicated. (**b**) Cells with cytoplasm/nucleus staining were counted and the ratio in the overall cell count was calculated (N = 300). The data are represented as mean ± SD. ^***^P < 0.001 (*P* = 0.00014).

We next investigated the cellular localization of β-catenin in tumors derived from PC9 or PC9M2 cells in a mouse xenograft model. Parental PC9 cells or gefitinib-resistant PC9M2 cells were injected subcutaneously into the flanks of severe combined immunodeficient (SCID) mice. We confirmed that gefitinib treatment significantly inhibited *in vivo* tumor growth derived from PC9 cells but not that of PC9M2 cells (Fig. 4). After 3 weeks, the tumors were resected and were analyzed by HE staining and by immunohistochemistry using anti-β-catenin antibodies and control immunoglobulin G (IgG) (Fig. 5a). Many cuboidal epithelial cells were tightly packed in PC9 cell-derived tumor tissues, whereas PC9M2 cell-derived tumor tissues were morphologically undifferentiated and contained many tumor cells with cell bodies and nuclei of irregular size, as well as a stroma-like component. β-catenin was strongly stained in the plasma membrane in the cuboidal epithelial cells in the tumor tissues derived from PC9 cells. In contrast, in the tumor tissues derived from PC9M2 cells, β-catenin was localized in the cytoplasm in most cells and there were a few cells that displayed positive staining in both the cytoplasm and the nucleus. No β-catenin staining was detected in the stroma-like component in PC9M2 cell-derived tumor tissues. We counted the number of cells displaying β-catenin staining in the cytoplasm or/and nucleus. We found that there were significantly more cells in which β-catenin was localized in the cytoplasm/nucleus in PC9M2 than in PC9 cells (Fig. 5b). These results suggest that the β-catenin in PC9M2 cell-derived tumors is more highly activated than that in PC9 cells.

### Plasma membrane localization of β-catenin is reduced in lung cancer tissues derived from gefitinib-resistant patients

**Figure 6:**
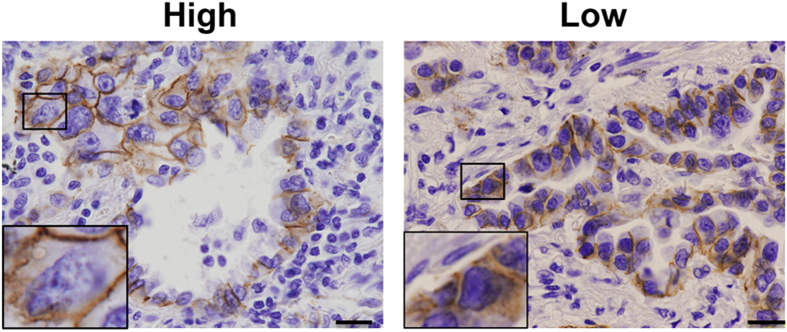
β-catenin expression pattern in EGFR mutant lung cancer patient tissues. The tumors derived from patients who harbored EGFR mutation and treated with EGFR-TKIs were stained using β-catenin antibody. The patients were categorized into three groups (“Negative”, “High” and “Low”) according to the population of the cells expressed membrane-localized β-catenin. The patients whose tumor contained more than 40% cells where β-catenin was stained in the plasma membrane were categorized into “High”, whereas the patients whose tumor contained less than 40% cells were categorized into “Low”. Representative images of tissues with “High” and “Low” categories. Scale bar = 20 μm. The insets are higher-magnification images of the circled areas. The inset in the left panel shows cells with plasma membrane staining. The inset in the right panel shows cells with very faint or no plasma membrane staining.

Finally, we immunohistologically analyzed β-catenin localization in lung cancer tissues harboring EGFR TKI-sensitive mutations that were derived from patients who were subsequently treated with gefitinib. The clinicopathological characteristics of the patients are shown in the Supplementary Table 1. We found 29/32 cancer tissues in which β-catenin staining was positive in the membrane, cytoplasm or nucleus. We found the staining in either the membrane, cytoplasm or nucleus was very weak in 3 samples. We regarded them as negative staining and omitted them from the analysis. Nuclear staining was not clearly detected in many samples, while the staining in the membrane was clearly detected. Because β-catenin localized in the plasma membrane appears to bind to α- and E-cadherin, activation of the β-catenin pathway might be inhibited^22^. We hypothesized that a large population of the cells expressing membrane-localized β-catenin might reflect weak β-catenin activity in the tumor. We counted the number of cells where β-catenin was stained in the plasma membrane. The patients whose tumor contained more than 40% cells where β-catenin was stained in the plasma membrane were categorized into “High” (Fig. 6, left panel). Whereas the patients whose tumor contained less than 40% where β-catenin was stained in plasma membrane were categorized into “Low”. In these patient tumors, β-catenin was stained in cytoplasm and/or nucleus (Fig. 6, right panel). As a control, we showed the image with negative β-catenin staining (Fig. S1).

We then correlated the β-catenin staining pattern with the clinical effects of gefitinib-treatment, which were classified as follows: “partial response”; patients who responded partially (8 cases), “no response”; patients who did not respond significantly (21 cases)^35^. As shown in Table 1, the “Low” pattern was more evident in lung cancer tissues of no-response cases than in those of partial-response cases and the “High” pattern was more evident in lung cancer tissues of partial-response cases than in those of no-response cases (*P* = 0.049). These results suggest that localization of β-catenin was related with gefitinib sensitivity in lung adenocarcinoma patients.

## Discussion

In this study, we established the gefitinib resistant cell line, PC9M2 that was derived from PC9 cells, which harbor an EGFR mutation. Neither the EGFR T790M mutation nor MET amplification was detected in the PC9M2 cells, suggesting the existence of other mechanisms of resistance to gefitinib in this gefitinib-resistant cell line. By comparison of the gene expression pattern of PC9 and PC9M2 cells, we found that Wnt/β-catenin-related genes were upregulated in PC9M2 cells. We confirmed that the β-catenin pathway is indeed activated in PC9M2 cells by determination of enhanced phosphorylation of Akt and GSK3α, cytoplasmic localization of β-catenin and activation of *Axin2* gene transcription. We next demonstrated that downregulation of β-catenin using siRNA or an inhibitor of β-catenin activity, restored gefitinib sensitivity to PC9M2 cells, indicating that β-catenin activation conferred gefitinib resistance to the cells. Furthermore, we examined the activation of β-catenin *in vivo* using a mouse xenograft model. Plasma membrane localization of β-catenin was more prominent in the tumor derived from PC9 cells than in that derived from PC9M2 cells, whereas cytoplasmic and nuclear β-catenin localization were more evident in the tumor derived from PC9M2 cells than in that derived from PC9 cells. These observations indicate that β-catenin activation was enhanced in PC9M2 cells compared with PC9 cells *in vivo*.

The major genetic abnormalities that are frequently observed in NSCLC are abnormalities in K-Ras and in receptor tyrosine kinases such as EGFR^10^,^36^, ALK^37^and RET^38^,^39^,^40^. All of these mutations lead to activation of Akt. Our data are to demonstrate that enhancement of the β-catenin pathway could be a common mechanism of resistance to EGFR TKIs. We observed that activation of Akt is increased in PC9M2 cells compared to PC9 cells. It is known that activated Akt phosphorylates GSKα^32^, leading to GSKα inactivation and that inhibition of GSK activity results in activation of β-catenin. Thus, cells harboring higher activation levels of Akt may survive even after long exposure to gefitinib treatment and activate β-catenin by inhibiting GSK activity. One possibility is that a minor population of tumor cells, in which the β-catenin pathway is already activated, might survive long-term exposure to gefitinib and ultimately become the major population. Another possibility is that novel mutations or epigenetic mechanisms might have occurred in a few cells due to the stress of gefitinib treatment and that these cells expanded to become the major population, since they were resistant to gefitinib. These two possibilities are not mutually exclusive.

There are many reports that Wnt/β-catenin pathways play important roles in the maintenance of cancer stem cells^22^,^41^,^42^. One of the typical characteristics of cancer stem cells is resistance to various kinds of cancer treatment. It is possible that a minor population of cancer stem-like cells in the PC9 population survive gefitinib treatment and ultimately become the major population in PC9M2 cells. If so, PC9M2 cells might have some cancer stem cell-properties. Given that the β-catenin pathway is important for cancer stem cells, it would be interesting to examine if the Akt-β-catenin pathway contributes to resistant mechanisms not only to other molecular targeted drugs but also to other conventional chemotherapy or radiotherapy.

Since the cellular localization of β-catenin should at least in part reflect the activity of the β-catenin pathway, we examined whether β-catenin localization in lung adenocarcinoma tissues with an EGFR mutation is associated with primary resistance to gefitinib. We observed that cytoplasmic localization of β-catenin is associated with lung adenocarcinoma tissues derived from primary gefitinib-resistant patients (no response cases) rather than with those from gefitinib-sensitive patients (partial response cases). We did not observe clear nuclear localization of β-catenin, which might be because β-catenin activation in the patient samples was not as strong as that in PC9M2 cell-derived tumors. However, it is possible that the β-catenin pathway is more highly activated in lung cancer tissues derived from patients who are resistant to EGFR-TKI treatment than in tissues derived from patients in whom the EGFR-TKI treatment is effective. It has been demonstrated that β-catenin activation is critical for tumorigenesis in an ErbB2/HER2-mediated mouse mammary tumor model^43^. In this report, mouse tumor tissues overexpressing ErbB2 showed nuclear localization of β-catenin. However, in human breast cancer tissues overexpressing ErbB2, β-catenin is mainly localized in the cytoplasm rather than in the nucleus. These observations suggest that β-catenin activation in the patient samples was not as strong as that in the mouse mammary tumors; this observation is in agreement with our own findings.

Our results raise the possibility that drugs targeted against the β-catenin pathway may be effective for patients whose lung cancer tissues display cytoplasmic localization of β-catenin. Since part of the adenocarcinoma tissues of gefitinib-sensitive patients display cytoplasmic localization of β-catenin, these patients may also be sensitive to drugs targeted against the β-catenin pathway. Obviously, it would be important to analyze β-catenin localization in lung cancer tissues derived from patients with acquired resistance to gefitinib. However, we did not have a large enough number of lung cancer tissues for such an analysis in the present study. Analysis of a large number of samples might be needed to validate the effect of drugs targeted against the β-catenin pathway on acquired resistance to gefitinib in patients.

## Methods

### Materials

Recombinant human EGF was purchased from Merck Millipore (Darmstadt, Germany). Gefitinib, MM-2206 and ICG-001 were purchased from Selleck chemicals (Houston, TX, USA).

### Cell culture and establishment of gefitinib resistant cell lines

PC9 cells were maintained in RPMI1640 medium supplemented with 10% FBS, 100 U/mL penicillin and 100 mg/mL streptomycin, at 37 °C and 5% CO2. To establish gefitinib resistant cell lines, parental PC9 cells were treated with gefitinib at concentrations of 20–100 nM for 14–28 days, 5–20 nM for 14–28 days and 100 nM for a range of exposure durations, yielding approximately 500 survived clones. We selected PC9-derived cells that showed a difference in gefitinib sensitivity but not in morphology or cell growth rate. Among the 500 clones that survived long-term exposure to gefitinib, we selected clones based on MTT assay. We treated the cells with 0, 0.1 and 1 μM of gefitinib, cultured them for 7 days and selected the clones that showed resistance to gefitinib, which resulted in a total of 179 clones (Fig. S2 and data not shown). Next, we selected the clones with PC9 cell-like morphology consisting of a mixture of attached and floating cells. A total of 12 clones were obtained in this manner, which we designated as “PC9M” lines (Fig. S3 and data not shown). We confirmed that none of these clones carried the EGFR Y790M mutation or showed MET amplification (Fig. 1b,c and data not shown). Finally, we cultured the cells and counted them daily for 4 days. We found that PC9M1, 2 and 3 cells grew at a similar rate as PC9 cells (Fig. S4 and data not shown). We selected PC9M2 cells for further analysis.

### Sanger sequencing and quantitative RT-PCR

RNA isolation, cDNA preparation and sequencing were described previously^38^. Primers used for analysis of *EGFR* were 5′- TGT AAA ACG ACG GCC AGT CGA AAG CCA ACA AGG AAA TCC-3′ (forward) and 5′- CAG GAA ACA GCT ATG ACC ATT CCA ATG CCA TCC ACT TGA T-3′ (reverse). To quantitatively analyze the *Axin2* mRNA expression level, quantitative real time PCR (qRT-PCR) was performed using a TaqMan probe as previously described in detail^44^. TaqMan probes for *Axin2* (Hs00610344_m1) and for 18S ribosomal RNA (Hs99999901_s1) were purchased from Life technology (Carlsbad, CA).

### Microarray experiments

Each cell line was starved for 48 h prior to the microarray experiments. Total RNA was then isolated from each line at 26 time points over a 24 h period. Hybridization was carried out on a 44K Agilent Whole Human Genome Oligo Microarray (G41112F) (Agilent Technologies). Microarrays were scanned using a dynamic autofocus microarray scanner (Agilent DNA Microarray Scanner, Agilent Technologies) under default parameters (Green PMT was set to 100% and the scan resolution was set to 5 μm). Feature Extraction Software v9.1 (Agilent Technologies) was used to obtain a raw signal value and a quality flag (Present [P], Marginal [M], or Absent [A]). After aggregating all the scanned signals for the different time points, cell lines and treatments into a single data matrix, quantile normalization was applied to remove the between-experiment distributional biases due to assay artifacts by forcing the same percentile values on all of the processed data. To assign a unique probe ID to each gene that is inherently spanned by multiple probes, we selected a probe with the maximal variance across the overall data points. Outliers were further processed by comparing the distance between the data points and smoothing spline curve fitted under a degree of freedom and then replacing all of the data points lying above a threshold on the distance to the smoothed value. Finally, a gene filtering procedure based on a specifically customized statistical test (details are omitted) excluded genes that maintained expression levels for 24 h that were lower than a properly controlled threshold. These preprocessing procedures made up the eight time course microarray data from 17,654 genes.

To identify pathways involved in the maintenance of gefitinib resistance, we focused on genes whose expression was specifically elevated in the gefitinib resistant PC9M2 cells in spite of the presence or absence of gefitinib-treatment or EGF-treatment. For each treatment type, a paired two-sample *t*-test was performed on the two sets of time-course measurements. For gene *i* and treatment *j*, the expression values of the parental PC9 and PC9M2 cells, *Y*_*ijt*_^PC9^ and *Y*_*ijt*_^PC9M2^, consisted of paired samples over the 26 time points (*t* = 1, ^…^, 26). A one-sided test was used to assess the increased expression of gene *i* in the PC9M2 cells. We then controlled the false discovery rate of the multiple testing over 17,654 genes at *q*-values < 0.05^45^. This analysis screened out four sets of 5,876, 5,991, 6,396 and 6,164 differentially-upregulated genes (denoted by *G*_1_, *G*_2_, *G*_3_ and *G*_4_ respectively) in the PC9M2 cells treated with control (*j* = 1), gefitinib (*j* = 2), EGF (*j* = 3) and both EGF and gefitinib (*j* = 4), respectively.

To further narrow-down the identified gene sets, we selected genes that were differentially regulated by gefitinib treatment in the parental PC9 and gefitinib resistant PC9M2 cells. Within each cell type, the difference in gene expression values was taken over the time points between the control and gefitinib-treated data sets, *d*_*it*_^PC9^ = *Y*_*i1t*_^PC9^– *Y*_*i2t*_^PC9^ and *d*_*it*_^PC9M2^ = *Y*_*i1t*_^PC9M2^– *Y*_*i2t*_^PC9M2^. To assess the difference in expression of each gene in the drug response between the parental and PC9M2 cells, a two-sided paired *t*-test was carried out with the two differentiated time-course profiles, *d*_*it*_^PC9^ and *d*_*it*_^PC9M2^ (*t* = 1, ^…^, 26). The produced *p*-values were converted to *q*-values. Controlling the false discovery rate at a *q*-value < 0.05, we obtained a set *G*_5_ of 6,421 genes that responded differently to gefitinib treatment across the cell types. The intersection of the five gene sets, *G*_1_, *G*_2_, *G*_3_, *G*_4_, *G*_5_, then resulted in 1,696 statistically significant genes that consisted of a set *G*.

Finally, we investigated enrichment of Gene Ontology (GO) terms in the gene set *G*. An overrepresentation test based on hypergeometric distribution^46^ was performed on each GO term that appeared at the fourth level of the directed acyclic graph in the class of ‘*biological process*’. This analysis detected strong enrichment of signals of the Wnt/β-catenin pathway, as the term GO:0016055 ‘Wnt receptor signaling pathway’ was ranked 9th (P = 0.003) in the list of 1,656 fourth-level ontology terms that were arranged according to increasing order of the *p*-values (Supplementary Table 2). Microarray data are available at the Gene Expression Omnibus website (www.ncbi.nlm.nih.gov/geo) under the accession number GSE34228.

### Western blotting

Cells were lysed in a lysis buffer (20 mM HEPES-NaOH (pH 7.5), 3 mM MgCl_2_, 100 mM NaCl, 1 mM DTT, 1 mM Na_3_VO_4_, protease inhibitor cocktail, phosphatase inhibitor-cocktail and 0.5% NP-40). Protein expression levels of EGFR, HER2 and HER3 were examined by immunoblotting using anti-EGFR (MBL), anti-HER2 (Cell Signaling Technology, Beverly, MA), anti-HER3 (Millipore), anti-phospho-EGFR (Cell Signaling Technology) and anti-phospho-HER2 (Cell Signaling Technology) antibodies respectively and anti-β-actin antibody (Millipore) as a loading control. Total and phosphorylated GSK3α were detected using anti-GSK3α (Cell Signaling Technology) and anti-phospho GSK3α (Ser21) (Cell Signaling Technology) antibodies at a 1:1000 dilution. Nuclear and cytosolic fractions were prepared as follows. Cells were treated with or without 1 μM gefitinib for 1 hour and were lysed in buffer A (10 mM HEPES-KOH (pH 7.8), 10 mM KCl, 0.1 mM EDTA, 0.1% NP-40, 1 mM DTT, 0.5 mM PMSF and protease inhibitor cocktail (Nacalai Tesque, Japan)). Cell lysate was centrifuged at 2,000 × *g* for 2 min at 4 °C. The pellets were resuspended in buffer C (50 mM HEPES-KOH (pH 7.8), 420 mM KCl, 0.1 mM EDTA, 5 mM MgCl_2_, 2% glycerol, 1 mM DTT, 0.5 mM PMSF and protease inhibitor cocktail) and rotated at 4 °C for 30 min. Nuclear extracts were prepared by centrifugation at 15,000 × *g* for 15 min at 4 °C. The expression of β-catenin in nuclear or cytosolic fractions was detected by immunoblotting using an anti-β-catenin (Cell Signaling Technology) and anti-HistonH3 (Cell Signaling Technology #4499) antibodies and horseradish peroxidase-conjugated secondary antibodies specific to mouse or rabbit IgG (BD bioscience). Signals were detected by Immobilon (Merck Millipore).

### RNA interference

The β-catenin siRNA and control siRNA were purchased from Cell Signaling technology PC9 or PC9M2 cells (2.5 × 10^4^ cells) were plated in a 12-Fwell plate and transfected with 50 nM siRNA using Lipofectamine® RNAiMAX Transfection Reagent according to the manufacturer’s protocol. At 72 h after transfection, cells were analyzed as described in the text.

### Cell viability assay

PC9 cells or PC9M2 cells were plated onto 96-well plate. Cells were treated with gefitinib and/or ICG-001 at the indicated concentrations. After 72 hrs treatment, the cell viability was measured using CellTiter 96 AQuesou One Solution Cell Proliferation Assay (Promega, USA).

### Mouse xenograft studies and immunohistochemical analysis

PC9 cells or PC9M2 cells were injected subcutaneously into the flanks of 6-weeks-old female SCID mice (5 × 10^6^ cells/mouse) (N = 6). From 6 days after injection, gefitinib (10 mg/kg/day) or vehicle was administrated orally everyday. After 2 ~ 3 weeks, the tumor was resected and immunohistochemically analyzed. Tumor volume was measured 3 times a week using the following formula: *V* = 1/2(*L* × *W*^2^), where *L* equals length and *W* equals width. Tumors were fixed in formalin, embedded in paraffin and then stained with hematoxylin-eosin (HE). Tissue sections were deparaffinized in xylene, rehydrated in graded ethanol and subjected to antigen retrieval. Immunohistochemistry was performed using the β-catenin antibody (Code M3539: Dako, Glostrup, Denmark) at a 1:1000 dilution and signal was detected using EnVision (DAKO ChemMate). All experiments were performed in accordance with the protocols approved by the Institutional Animal Care and Use Committee at Institute of Medical Science, University of Tokyo and Kanazawa University.

### Analysis of human lung cancer tissues

Thirty-two tumor specimens with EGFR mutations were obtained from NSCLC patients, all of whom provided written informed consent, at Kanazawa University, Osaka City University, Tokushima University Hospital, Osaka Medical Center for Cardiovascular Diseases and the National Cancer Center Hospital in Japan. This study was approved by the Institutional Review Board of Institute of Medical Science, University of Tokyo, Kanazawa University, Tokyo Medical University, Osaka City University, National Cancer Center Hospital, Osaka Medical Center for Cancer and Cardiovascular Diseases, Institute of Health Bioscience and Hokkaido University. The study was carried out in accordance with the guidelines of the Medical Ethics committees of Institute of Medical Science, University of Tokyo and Kanazawa University. Informed consent was obtained from all subjects.

Patient characteristics are shown in Supplementary Table 1. *EGFR* exon 19 deletion and EGFR L858R or G719X point mutations were detected in these tumors. Gefitinib and erlotinib were given as the first EGFR-TKI to 23 and 9 patients, respectively.

Immunohistochemical staining for β-catenin was conducted on formalin-fixed, paraffin-embedded tissue sections (4 μm thick) of tumor specimens with microwave antigen retrieval in 0.01 M citrate buffer (pH 6.0). Mouse monoclonal antibody against β-catenin at 1:200 dilution was used as the primary antibody and EnVision/HRP Polymer Reagent and DAB (3, 3′-diaminobenzidine tetrahydrochloride) Liquid (Dako) were used for detection.

### Statistical analysis

Data in the graphs are represented as means ± standard deviation (S.D.). Statistical significance was assessed using Student’s *t*-test according to the variance between the two samples. Statistically significant differences are indicated with an asterisk (P < 0.05), double asterisks (P < 0.01) or with triple asterisks (P < 0.001) in the figures. We used Barnard’s test for analysis of the contingency table^47^,^48^.

## Additional Information

**How to cite this article**: Nakata, A. *et al.* Elevated β-catenin pathway as a novel target for patients with resistance to EGF receptor targeting drugs. *Sci. Rep.* **5**, 13076; doi: 10.1038/srep13076 (2015).

## Supplementary Material

Supplementary Information

## Figures and Tables

**Table 1:** Clinical relevance of β-catenin expression pattern to EGFR-TKI efficiency.

Response to EGFR-TKI	% of β-catenin positive tumor cells with homogenous plasma membrane staining	
	Low: 0–40%	High: 50–100%
Partial response	4/8 (25%)	6/8 (75%)
No response	14/21 (67.7%)	7/21 (33.3%)
