# Supplementary material for: Elevated β-catenin pathway as a novel target for patients with resistance to EGF receptor targeting drugs
Source: Sci Rep. 2015 Aug 13;5:13076. doi: 10.1038/srep13076 (PMC4535059; doi:10.1038/srep13076)
Supplement: Supplementary Information [file srep13076-s1.pdf]

Elevated β-catenin pathway as a novel target for patients with resistance to EGF receptor targeting drugs

Asuka Nakata[1,2], Ryo Yoshida[3], Rui Yamaguchi[4], Mai Yamauchi[2], Yoshinori Tamada[5], Andre Fujita[5], Teppei Shimamura[5], Seiya Imoto[5], Tomoyuki Higuchi[3], Masaharu Nomura[6], Tatsuo Kimura[7], Hiroshi Nokihara[8], Masahiko Higashiyama[9], Kazuya Kondoh[10], Hiroshi Nishihara[11], Arinobu Tojo[2], Seiji Yano[12], Satoru Miyano[4,5], Noriko Gotoh* [1,2]

[1] Division of Cancer Cell Biology, Cancer Research Institute, Kanazawa University

[2]Division of Molecular Therapy, Institute of Medical Science, University of Tokyo

[3] The Institute of Statistical Mathematics

[4]Laborabory of Sequence Analysis, Institute of Medical Science, University of Tokyo

[5]Laborabory of DNA information Analysis, Institute of Medical Science, University of Tokyo

[6] Department of Surgery, Tokyo Medical University

[7] Department of Respiratory Medicine, Graduate School of Medicine, Osaka City University

[8] Division of Internal Medicine and Thoracic Oncology, National Cancer Center Hospital

[9] Department of Thoracic Surgery, Osaka Medical Center for Cancer and Cardiovascular Diseases

[10] Department of Thoracic, Endocrine Surgery and Oncology, Institute of Health Bioscience

[11] Laboratory of Translational Pathology, Hokkaido University Graduate School of Medicine

[12] Division of Medical Oncology, Cancer Research Institute, Kanazawa University

Figure S1

Negative β-catenin staining

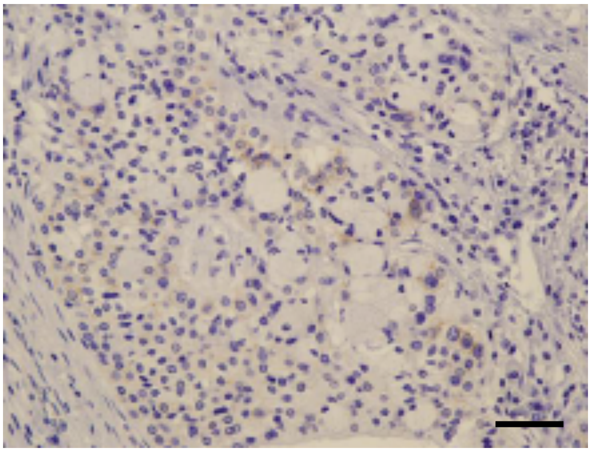

Figure S1
A representative image of negative β-catenin staining. Scale bar = 100 μm.

Figure S2

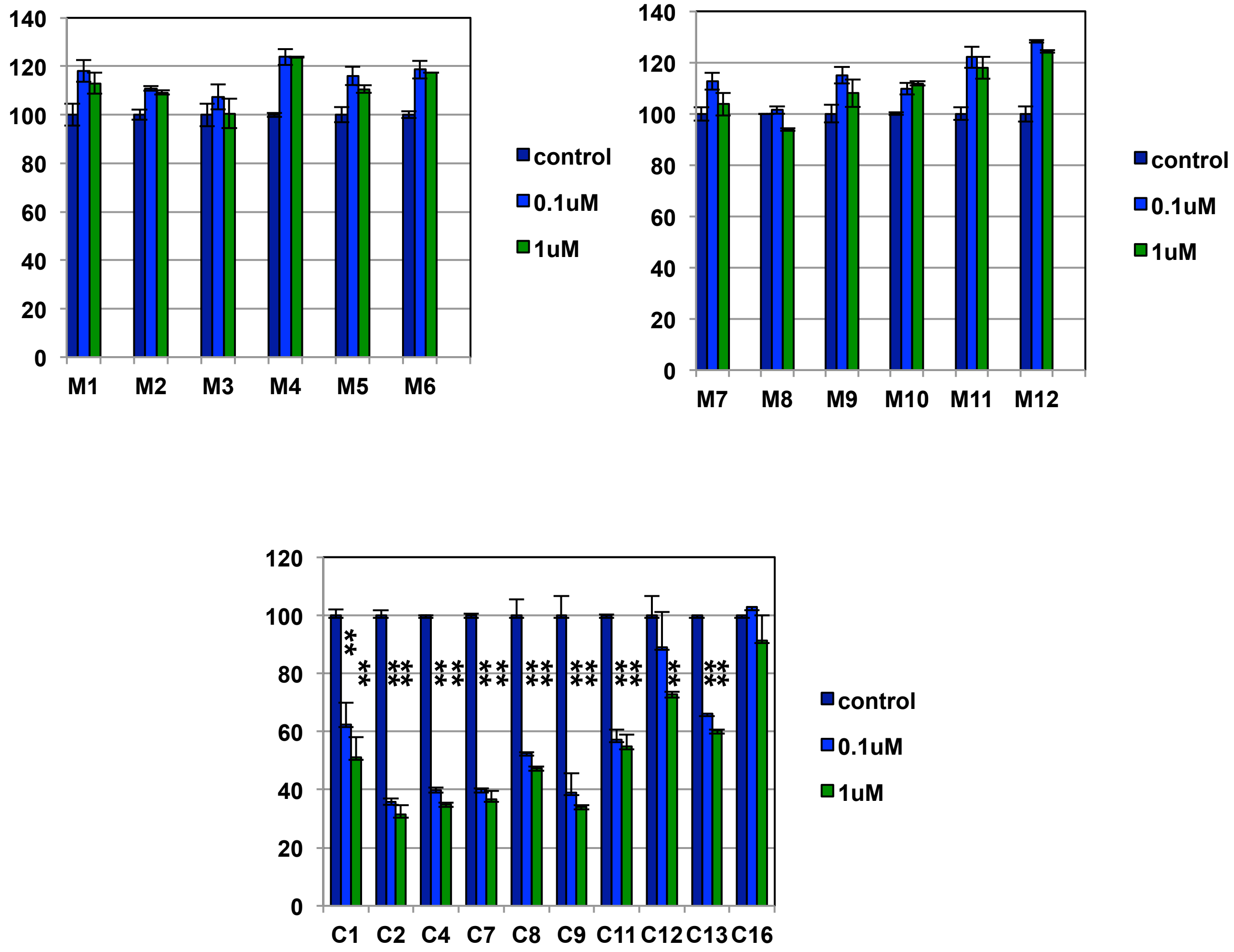

Figure S2

PC9M line cells are resistant to gefitinib in vitro. Cells were cultured under the treatment with or without gefitinib (0, 0.1μM and 1μM) for 7 days. Cell growth rates were analyzed by MTT assay. Data were represented as mean $\pm$ SD (N = 4). **, *P* < 0.01. PC9M line cells are indicated as M1-12 and PC9C line cells are indicated as C1-16.

Figure S3

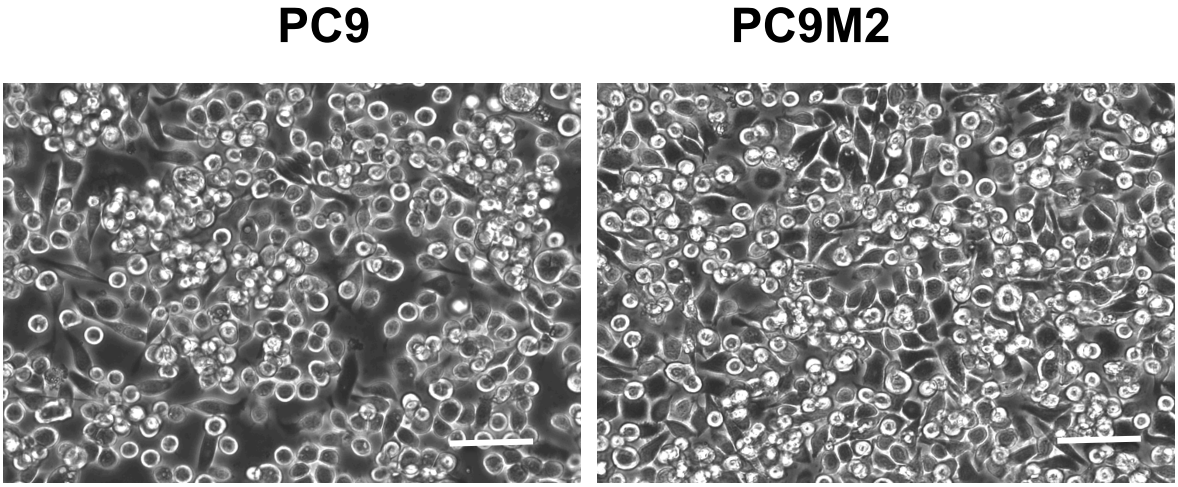

Figure S3
Morphology of PC9 cells and PC9M2 cells. PC9 cells and PC9M2 cells showed similar morphology with a mixture of attached cells and floating cells. Scale bar, 50μm.

Figure S4

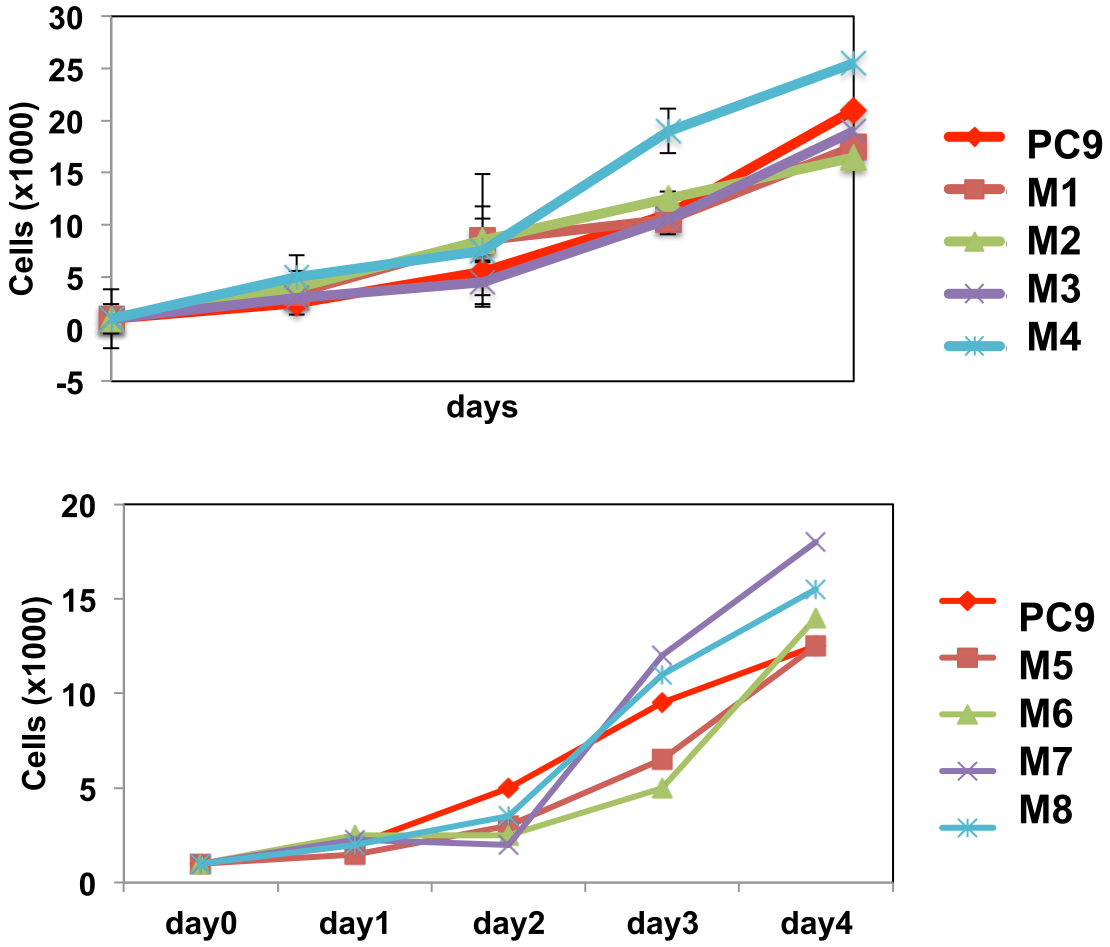

Figure S4
Growth curves of PC9 and PC9M lines. Cell growth rates were counted at each day. Data were represented as mean ± SD (N = 4). PC9M lines are indicated as M1-8.

Supplementary Table 1

Clinicopathological characteristics of the lung cancer patients

Number of patients		29
Age	Median	62
	Range	38-86
Gender	Male	12
	Female	17
Smoking hstory	Former/current smoker	10
	Never smoker	19
Histological type	Adeno	27
	Squamous	1
	Undifferentiated NSCLC	1
EGFR mutation status	Exon 19 deletion	10
	L858R	15
	Exon 19 deletion+L858R	1
	G719X	3
EGFR-TKI treatment	Gefitinib	20
	Erlotinib	9
Response to EGFR-TKI	Partial Response	8
	No Response	21

Supplementary Table 2

Gene Ontology (GO) analysis of the 1,696 genes whose expression levels were significantly elevated in PC9M2 cells

Rank	GO term	Pvalue	Number of genes	Term in 'biological process'
1	GO:0070254	0.000457064	5	mucus secretion
2	GO:0035987	0.000497205	16	endodermal cell differentiation
3	GO:0045444	0.000788908	114	fat cell differentiation
4	GO:0046325	0.002712138	7	negative regulation of glucose import
5	GO:0042384	0.002726353	62	cilium assembly
6	GO:0001706	0.002938474	26	endoderm formation
7	GO:0046627	0.003232355	21	negative regulation of insulin receptor signaling pathway
8	GO:0048854	0.003232355	21	brain morphogenesis
9	GO:0016055	0.0035145	259	Wnt receptor signaling pathway
